# Supplementary material for: SARS-CoV-2 Omicron BA.1 Variant Infection of Human Colon Epithelial Cells
Source: Viruses. 2024 Apr 19;16(4):634. doi: 10.3390/v16040634 (PMC11055019; doi:10.3390/v16040634)
Supplement: Supplementary file 1 [file viruses-16-00634-s001.zip › viruses-2837252-supplementary.pdf]

Supplementary Materials

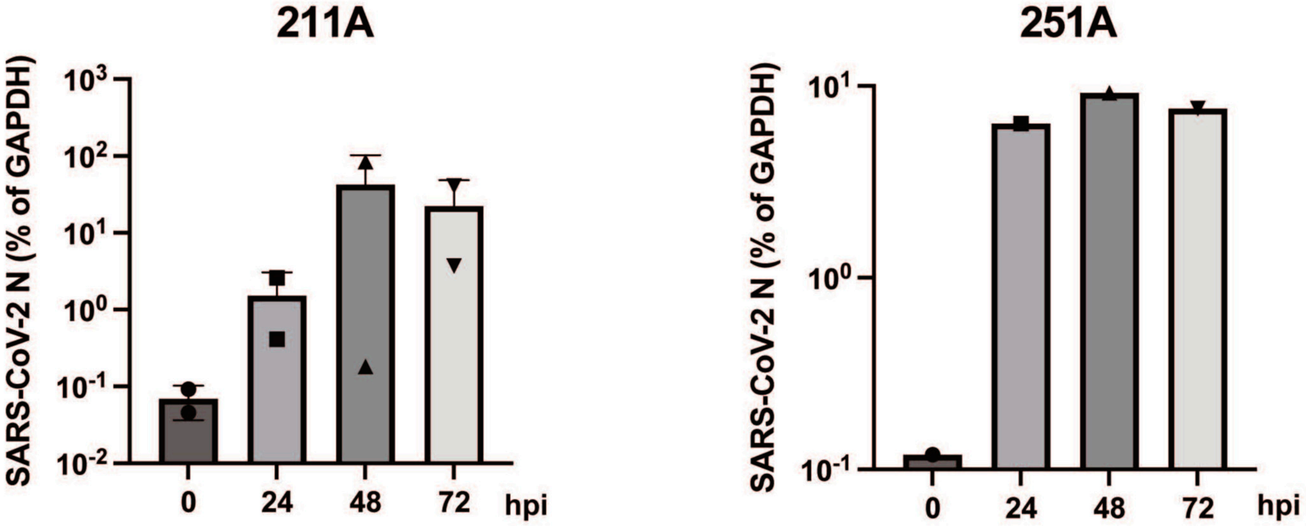

Supplemental Figure S1. Kinetics of SARS-CoV-2 replication in human colonoids. Colonoid lines in 2D transwell monolayers derived from two donors were infected with indicated SARS-CoV-2 variants at an MOI of 0.01. RNA was harvested at 24, 48, or 72 hours post infection and SARS-CoV-2 N level was quantified by RT-qPCR and normalized to GAPDH.

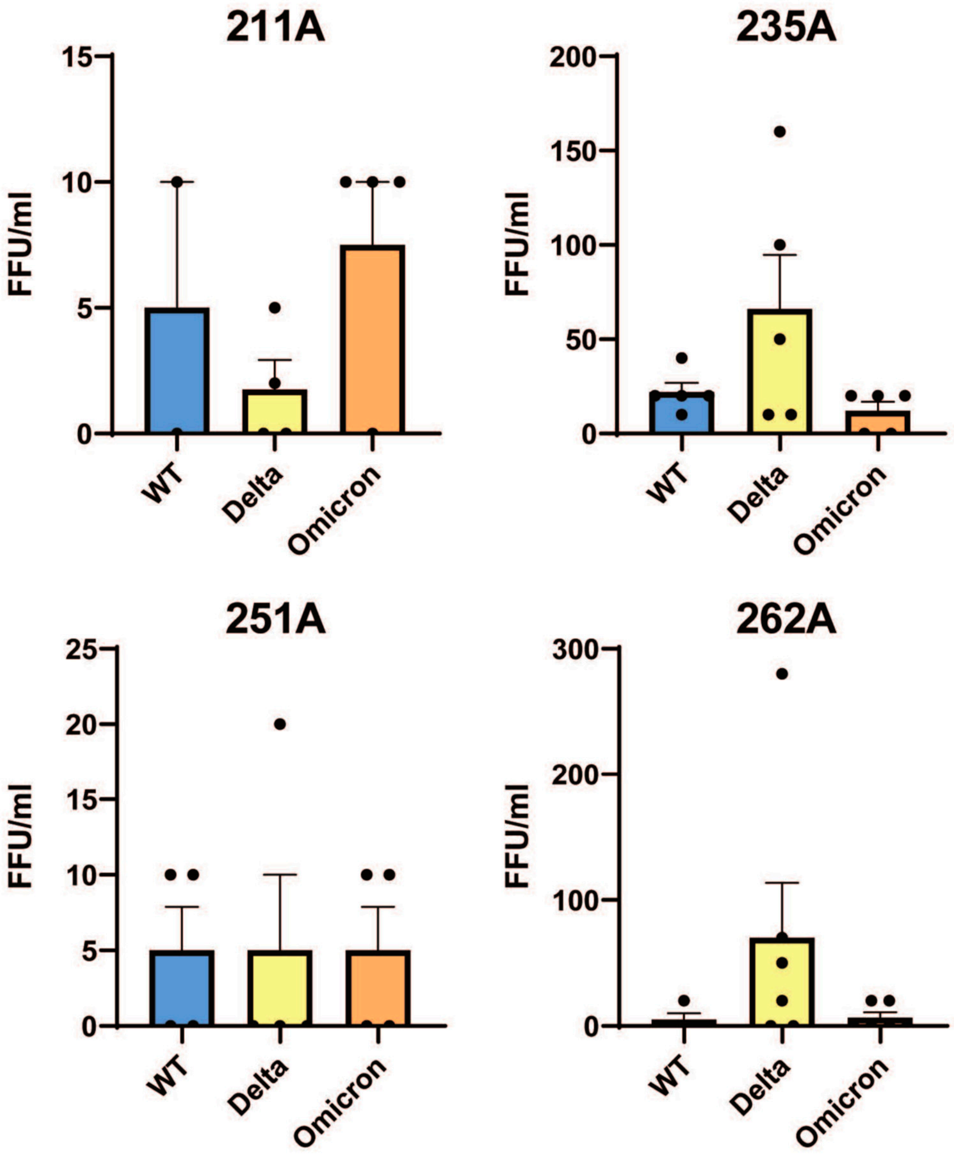

Supplemental Figure S2. Production of virus progeny from multiple SARS-CoV-2 variants. Quantification of infectious viral particles in the supernatants collected from the apical compartments of corresponding transwells at 24 hours post infection by a focus forming unit assay.

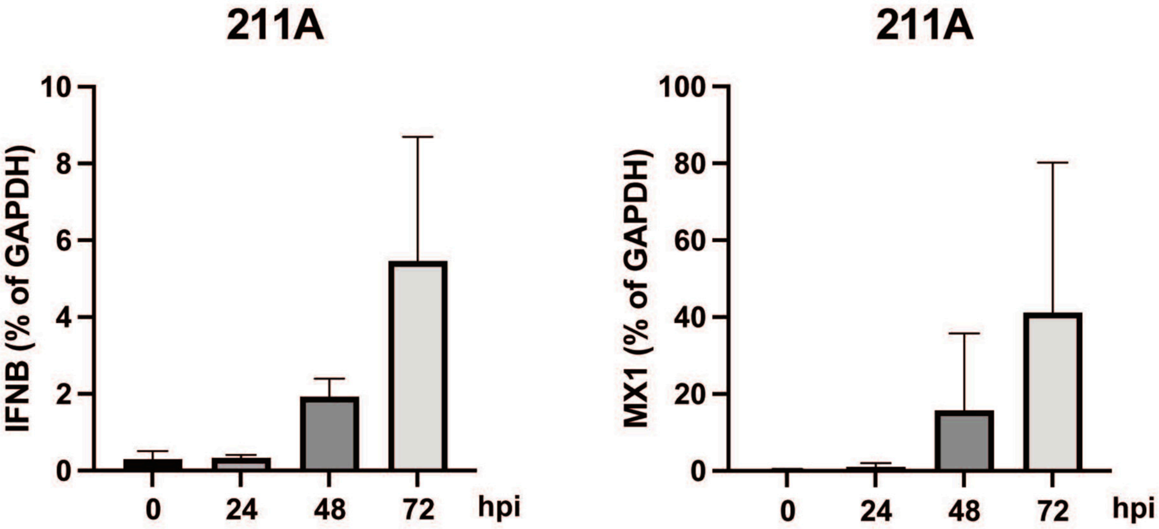

Supplemental Figure S3. Temporal dynamics of type I IFN expression. IFNB and MX1 mRNA levels were quantified by RT-qPCR and normalized to GAPDH in colonoids infected with WA1 and harvested at 24, 48, and 72 hours post infection. (Mean with SEM).

Table S1. Virus sequencing results: Spike Mutations in SARS-CoV-2 variants sequenced by NovaSeq.

Omicron B.1.1.529	Delta B.1.617.2	WT WA1
T95I, G142D, V143del, Y144del, Y145del, N211I, L212del, G339D, S371L, S373P, S375F, K417N, N440K, G446S, S477N, T478K, E484A, Q493R, G496S, Q498R, N501Y, Y505H, T547K, D614G, H655Y, N679K, P681H, N764K, D796Y, N856K, Q954H, N969K, L981F, D1146	T19R, G142D, E156G, F157del, R158del, L452R, T478K, D614G, P681R, F855S, D950N, C1235F	No spike mutations

Table S2. qPCR Primers and Probes.

Primer name	Sequences
SARS-CoV-2 N	Forward: ATGCTGCAATCGTGCTACAA<br>Reverse: GACTGCCGCCTCTGCTC<br>Probe: FAM/TCAAGGAACAACATTGCCAA/TAMRA
Human GAPDH	Forward: GGAGCGAGATCCCTCCAAAAT<br>Reverse: GGCTGTTGTCATACTTCTCATGG
IFNL3	Forward: TAAGAGGGCCAAAGATGCCTT<br>Reverse: CTGGTCCAAGACATCCCCC
IFNB	Forward: ATGACCAACAAGTGTCTCCTCC<br>Reverse: GGAATCCAAGCAAGTTGTAGCTC
MX1	Forward: GTGGCTGAGAACAACCTGTG<br>Reverse: GGCATCTGGTCACGATCCC

Disclaimer/Publisher's Note: The statements, opinions and data contained in all publications are solely those of the individual author(s) and contributor(s) and not of MDPI and/or the editor(s). MDPI and/or the editor(s) disclaim responsibility for any injury to people or property resulting from any ideas, methods, instructions or products referred to in the content.

Table S3. Background of the colonoids donors.

**Patient ID**	**Patient Diagnosis (N = non-IBD/healthy, CD = Crohn's, UC = ulcerative colitis, FAP = Familial**	**Center Biopsy Collected At**	**Intestinal region**	**Age @ Access**	**Sex 1=Male 2=Female**	**Race 1=White 2=Black 3=Asian 4=Hispanic 5=Multiple/Other 6=Native American**	

	**aden. Poly.**						
H211	N	WashU	Rectum	55	2	2	
H235	N	WashU	Rectum	51	2	2	
H251	N	WashU	Rectum	63	1	1	
H262	N	WashU	Rectum	56	2	1	
